# New Preventive Strategy against Oral Biofilm Formation in Caries-Active Children: An In Vitro Study

**DOI:** 10.3390/antibiotics12081263

**Published:** 2023-07-31

**Authors:** Ana Parga, Sabela Balboa, Paz Otero-Casal, Ana Otero

**Affiliations:** 1 Department of Microbiology and Parasitology, CIBUS-Faculty of Biology, Universidade de Santiago de Compostela, 15782 Santiago de Compostela, Spain; ana.parga.martinez@usc.es; 2 Department of Microbiology and Parasitology, Center of Cross-Disciplinary Research in Environmental Technologies (CRETUS), Universidade de Santiago de Compostela, 15782 Santiago de Compostela, Spain; 3 Department of Surgery and Medical-Surgical Specialties, Faculty of Medicine and Odontology, Universidade de Santiago de Compostela, 15782 Santiago de Compostela, Spain; 4 Unit of Oral Health, Centro de Saúde Santa Comba-Negreira, SERGAS, 15841 Santa Comba, Spain

**Keywords:** biofilm, microbiome, dental caries, quorum sensing, quorum quenching, acyl-homoserine lactones, *Streptococcus*, 16S rRNA gene amplicon sequencing

## Abstract

Quorum quenching (QQ) is the inhibition of bacterial communication, i.e., quorum sensing (QS). QS is a key mechanism in regulating biofilm formation and phenotype in complex bacterial communities, such as those found within cariogenic biofilms. Whereas QQ approaches were shown to effectively reduce biomass, knowledge of their impact on the taxonomic composition of oral polymicrobial biofilms remains scarce. Here, we investigate the effect of the QQ lactonase Aii20J on biomass production and taxonomical composition of biofilms. We collected supragingival plaque samples from 10 caries-free and 10 caries-active children and cultured them to generate in vitro biofilms. We describe significant biomass reductions upon Aii20J exposure, as assessed by crystal violet assays. Taxonomical profiling using 16S rRNA gene amplicon sequencing revealed no significant changes in bacterial composition at the genus level. Interestingly, at the species level Aii20J-treatment increased the abundance of *Streptococcus cristatus* and *Streptococcus salivarius*. Both *S. cristatus* and *S. salivarius* express pH-buffering enzymes (arginine deiminase and urease, respectively) that catalyze ammonia production, thereby potentially raising local pH and counteracting the biofilm’s cariogenic potential. Within the limitations of the study, our findings provide evidence of the biofilm-modulating ability of QQ and offer novel insights into alternative strategies to restore homeostasis within dysbiotic ecosystems.

## 1. Introduction

Caries is an oral disease that affects children and adults worldwide. The most recent estimate points to 520 million children suffering from caries in deciduous teeth and 2000 million people with caries in permanent teeth in 2019 [1]. When left untreated, caries can progress into more severe diseases, such as root canal infections, and eventually lead to bloodstream infections and endocarditis.

The classical specific plaque hypothesis highlighted the etiological role of bacterial taxa such as *Streptococcus mutans* as main players in dental caries. Nevertheless, the ecological plaque hypothesis describes caries as a polymicrobial disease resulting from an imbalance in the healthy oral biofilm [2,3]. This imbalance is commonly due to an increased intake of fermentable sugars and carbohydrates that bacteria metabolize, producing acidic compounds. A lowered pH maintained in time eventually surpasses the homeostatic mechanisms of the healthy oral biofilm through the selection of acidogenic and aciduric microorganisms, shifting towards a cariogenic biofilm [4,5,6]. Thus, it is now accepted that the functional activities within oral biofilms have a critical role in the progression of diseases, complementing that of the microbiome structure [3,7,8].

The search for novel strategies for the prevention and treatment of oral diseases has pointed out the possibility of interfering with cariogenic virulence factors without affecting bacterial viability [9,10,11,12,13]. One of these factors is bacterial communication, also known as quorum sensing (QS). The presence of the main QS pathways has been described in caries-associated species and species retrieved from carious lesions. Autoinducer peptides, typically used by gram-positives, have been identified in several *Streptococcus*, where QS mainly depends on the competence-stimulating peptide (CSP). Interference with the CSP signaling system in *S. mutans* has been shown to inactivate virulence factors and impair the persistence of this bacterium in the oral biofilm [14,15,16]. The autoinducer-2 (AI-2) has already been described in several oral pathogens, specifically *Streptococcus* [17,18]. Furthermore, the use of a cellular extract from *Tenacibaculum* sp. 20J is active against AI-2 reduced *S. mutans* biofilm formation in vitro, possibly by inhibiting AI-2 signaling [9]. Gram-negative *N*-acyl-homoserine lactones (AHLs) have been considered nonrelevant in the oral environment [11,17,19,20]. Nevertheless, AHLs have been found in saliva and tooth samples [12,21], and bacterial strains isolated from caries have been shown to produce AHLs [22,23]. Moreover, bacterial gene clusters predicted to be related to AHL biosynthesis have been described in the saliva metagenomes of caries-active children [24]. In addition, two studies on in vitro oral biofilms showed that exposure to AHLs and modified AHLs reduced lactic acid accumulation, even in the presence of fermentable sugars, without affecting bacterial growth [25,26]. More recently, the use of the quorum quenching (QQ) enzyme Aii20J, highly active against AHLs, was described as highly effective against biofilm formation in samples retrieved from patients with periodontal disease [27]. Interestingly, this previous study also found a great effect of the Aii20J enzyme on in vitro biofilms that were largely dominated by *Streptococcus* [27]. Altogether, this evidence points to the possibility of interfering with AHL-mediated communication mechanisms to modulate oral biofilm formation, even in bacterial populations where gram-negative bacteria are found only in low proportions.

This study aimed to characterize the effect of the QQ enzyme Aii20J on in vitro biomass production and changes in taxonomical composition of biofilm communities derived from supragingival plaque samples. The microbiome structure of the initial supragingival biofilms was investigated by 16S rRNA sequencing, and these supragingival samples were used to generate in vitro biofilms in the presence of the enzyme. The effects of Aii20J were investigated regarding biofilm mass (crystal violet (CV) assay) and microbial composition (Illumina sequencing of the 16S rRNA gene) of the in vitro biofilms. This study describes reductions in biomass of in vitro biofilms generated from samples of caries-free and caries-active children upon treatment with Aii20J. These observations were not accompanied by changes in the microbial diversity of the biofilms, as previously described for biofilms derived from adult patients with periodontal disease [27].

## 2. Results and Discussion

### 2.1. Microbial Composition of Supragingival Biofilm Samples from Caries-Free and Caries-Active Children

Supragingival biofilm was sampled from either caries-free or caries-active children (Table 1). To investigate the microbiome structure of these samples, their genomic DNA (gDNA) was extracted to sequence the V3–V4 hypervariable regions of the 16S rRNA gene.

**Figure 1:**
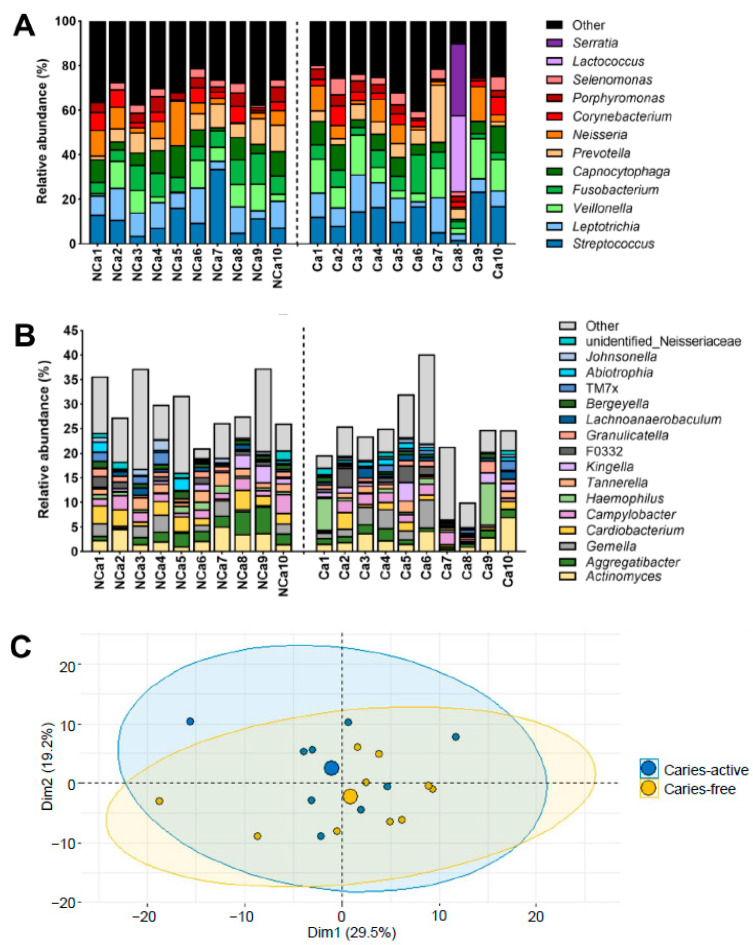
Relative abundance of the most abundant bacterial genera identified in the supragingival biofilm samples and principal components analysis (PCA) plots of their microbiota structure. Supragingival biofilms were sampled from the vestibular and lingual surfaces from caries-free (*n* = 10) and caries-active children (*n* = 10). (**A**). The relative abundance of the most relatively abundant genera identified in the samples is represented on the y-axis. Genera detected in lower relative abundance are grouped in the category “Other”. Supragingival samples from caries-free children (NCa) are shown on the left part of the x-axis, and samples from caries-active children (Ca) are on the right part of the x-axis. (**B**). The relative abundance of the genera grouped under the category “Other” are identified in relative abundances higher than 0.5%. Genera detected in relative abundances lower than 0.5% appear again under the category “Other”. (**C**). PCA plot of the 19 supragingival biofilm samples after removing sample Ca8 (*n* = 10 caries-free children, represented by yellow circles; *n* = 9 caries-active children, represented by blue circles).

Figure 1A,B shows the relative abundance of the most abundant genera identified in supragingival biofilm samples from caries-free and caries-active children. *Streptococcus* was found in relative abundances ranging from 2 to 34% among subjects. Such proportions are similar to those compiled in the Human Oral Microbiome Database (HOMD) based on the studies of Eren et al. [28] and of Segata et al. [29], with reported relative abundances of 15–30% for the genus *Streptococcus* in supragingival plaque samples. Besides *Streptococcus*, the most relatively abundant genera were *Leptotrichia*, *Veillonella*, *Fusobacterium*, *Capnocytophaga*, *Prevotella*, *Neisseria*, *Corynebacterium*, *Porphyromonas*, and *Selenomonas*. Yet, supragingival biofilms comprised a much higher number of taxa, with 38–71 total genera identified among samples. The less relatively abundant genera comprised proportions of up to 40% of the supragingival biofilms (Figure 1B). The only exception was sample Ca8, taken from a caries-active subject, in which the predominant genera were *Lactococcus* and *Serratia*, with relative abundances of 34% and 32%, respectively. Besides, this was the only sample in which these two genera were identified (Figure 1A). The principal components analysis (PCA) of the supragingival biofilm samples separated subject Ca8 from the others (Supplementary Materials Figure S1). Such differences in bacterial composition could not be explained by any clinical characteristic of subject Ca8, nor could we rule out an anomaly of a different kind. Its clear differentiation from similar samples (Supplementary Materials Figure S1) led to the exclusion of sample Ca8 from subsequent analyses. The microbiota structure of the supragingival biofilms was analyzed for the remaining 19 subjects (Figure 1C). The PCA displayed an almost complete overlap between samples from caries-free (yellow) and caries-active children (blue) (Figure 1C). Thus, within this collection of supragingival biofilm samples, the microbiota of caries-free and caries-active children are not significantly different. The observations presented in Figure 1C align with those of Belda-Ferre et al. [30], who did not find significant differences in the microbiome structure of supragingival plaque samples collected from healthy adults and patients with caries. It is relevant to note that Belda-Ferre et al. [30] used supragingival plaque samples taken from intact tooth surfaces, as in the present study, and not directly from the carious lesions. Interestingly, the protein profiles of those subgingival samples were clustered according to the caries status of the subjects [30]. Indeed, in recent years the emphasis has been put on the functional activities within oral microbial communities rather than on their microbiota distribution, as the former has been described to account for a greater impact on disease evolution [3,7].

**Figure 2:**
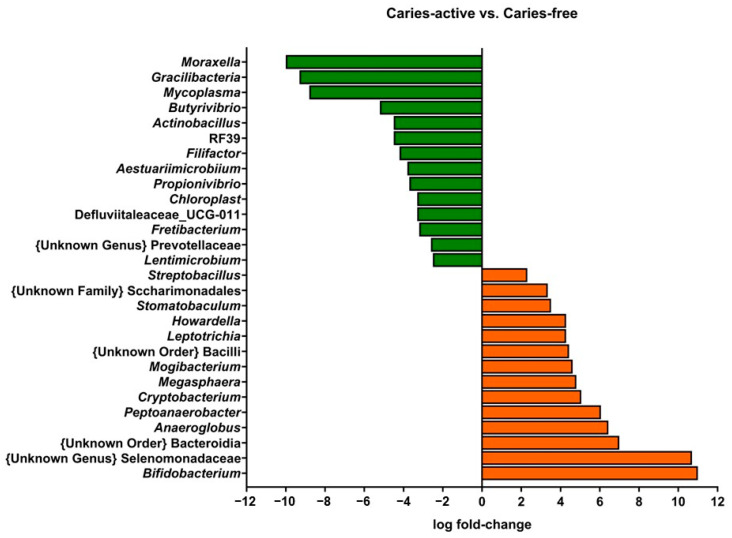
Linear discriminant analysis of the genera identified in supragingival biofilm samples from children with active caries, compared to caries-free children. Genera with log fold-change values lower than 2 (green bars) were considered as significantly reduced in caries-active subjects, and genera with log fold-change values higher than 2 (orange bars) were significantly increased in the same subjects.

Interestingly, when examining the microbial composition of the samples at the genus level with a linear discriminant analysis (LDA), genera present at very low relative abundances showed significant differential abundances between caries-free and caries-active subjects (Figure 2). Figure 2 shows positive fold-change values for genera that were more abundant in samples from children with active caries relative to caries-free children; whereas negative fold-change values represent the opposite case.

Genera classically associated with caries, such as *Streptococcus*, did not present relevant differences in relative abundance between caries-free and caries-active subjects, pointing to metabolic activities rather than specific taxon abundance as the most relevant parameter that differentiates cariogenic and healthy biofilms. Instead, low-abundance genera did present significant differences between caries-free and caries-active children. The genus *Bifidobacterium* was increased in caries-active biofilm samples. *Bifidobacterium* members can be highly acidogenic, initiating caries or aiding their progression [10,31]. Nevertheless, their increased presence in caries-active subjects can also be derived from a selection of acidogenic bacteria in an acidified environment. The genus *Moraxella* was significantly increased in caries-free samples compared to caries-active samples. This observation aligns with the study of Belda-Ferre et al. [32], in which they described a tendency of Gammaproteobacteria members to be more common in healthy subjects. The overall homogeneity of the principal genera identified among biofilms of subjects with different oral health statuses reinforces the need for proteomic or metatranscriptomic studies that allow the observation of differences in functional activities among samples and the description of the role of low-abundance taxa that are differentially represented in caries-free and caries-active subjects.

### 2.2. In Vitro Biofilm Formation from Caries-Free and Caries-Active Children

In vitro biofilms were generated using supragingival biofilm samples as inocula. Supragingival biofilms from either caries-free or caries-active children were inoculated in aerobic conditions in the Amsterdam Active Attachment biofilm model (AAA model) [26,33] in McBain medium and, in parallel, in McBain supplemented with 0.2% sucrose. The use of McBain medium permits the generation of biofilms containing up to 40% relative abundance of gram-negative taxa [27]. In contrast, the use of McBain supplemented with sucrose has been established as a successful in vitro setting for developing cariogenic polymicrobial biofilms [34].

**Figure 3:**
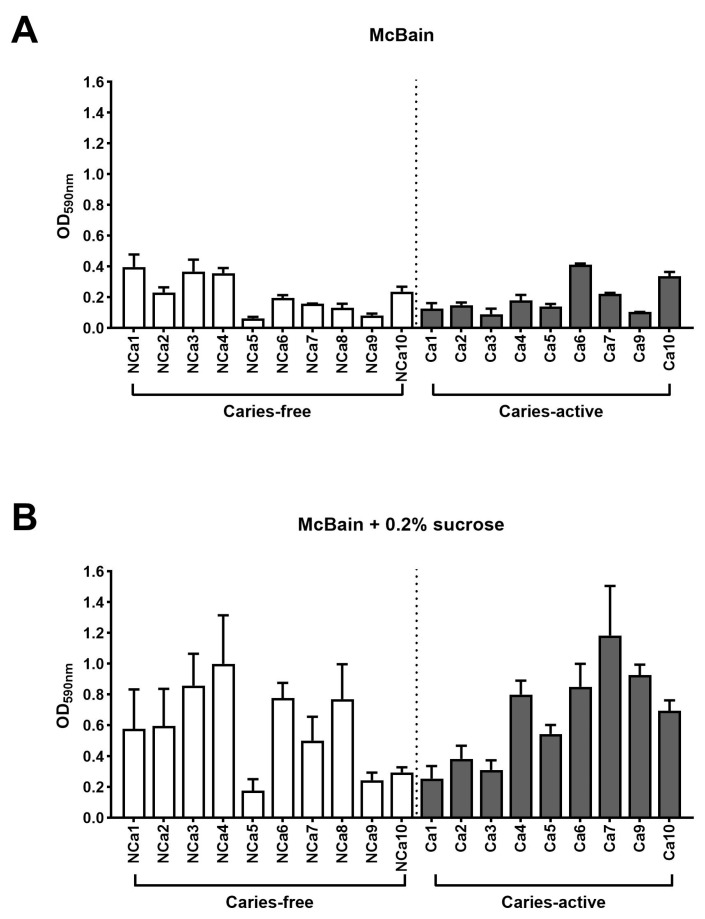
Quantification of in vitro supragingival-derived biofilms. Histograms represent the biofilm mass achieved by each sample without the Aii20J treatment (untreated controls), assessed by CV assays (y-axis). Results are represented as the mean of OD_590nm_ measurements, with error bars for standard deviation (*n* = 3). White bars represent biofilms from samples of caries-free children, whereas gray bars represent biofilms from samples of caries-active children (x-axis). Supragingival samples were grown in McBain medium (**A**), and McBain medium supplemented with 0.2% sucrose (**B**).

Figure 3 shows the values of total attached biofilm mass derived from supragingival samples grown without Aii20J (untreated controls). Biomass values, as assessed with CV assay, were heterogeneous among different subjects in both culture media used (Figure 3A,B). Comparison of the samples grouped according to the oral health status of the subjects, revealed no statistically significant differences between biofilm formation abilities of samples from caries-free and caries-active children in either of the culture media used (*p* = 0.8412 for biofilms grown in McBain; *p* = 0.4143 for biofilms grown in McBain-sucrose). These results showed that interindividual differences had a greater influence on the in vitro biofilm formation abilities of the samples than the oral health status of the subject. This observation aligns with previous reports on in vitro biofilms obtained from saliva and subgingival samples [27]. Here, the initial investigation of the microbial composition of the inocula used to generate biofilms did not reveal significant differences between subjects (Figure 1). Nevertheless, these microbial populations have different biofilm formation abilities upon in vitro cultivation (Figure 3). These results align with the increasing importance given to functional activities within microbial populations in addition to their bacterial composition [7,35]. Biomass quantification of biofilms grown in McBain ranged between 0.1 and 0.4 in absorbance (OD_590nm_) (Figure 3A); whereas biomass quantification of biofilms grown in McBain-sucrose was higher and ranged between 0.2 and 1.2 (Figure 3B). These results obtained with CV assay, subscribe to those of Janus et al. [34]. Using the same culture media as in the present study, they described that saliva-derived biofilms cultivated in the presence of sucrose achieved higher growth values and rates than biofilms cultivated without sucrose [34].

### 2.3. Effect of Aii20J on Supragingival-Derived Biofilms from Caries-Free and Caries-Active Children

**Figure 4:**
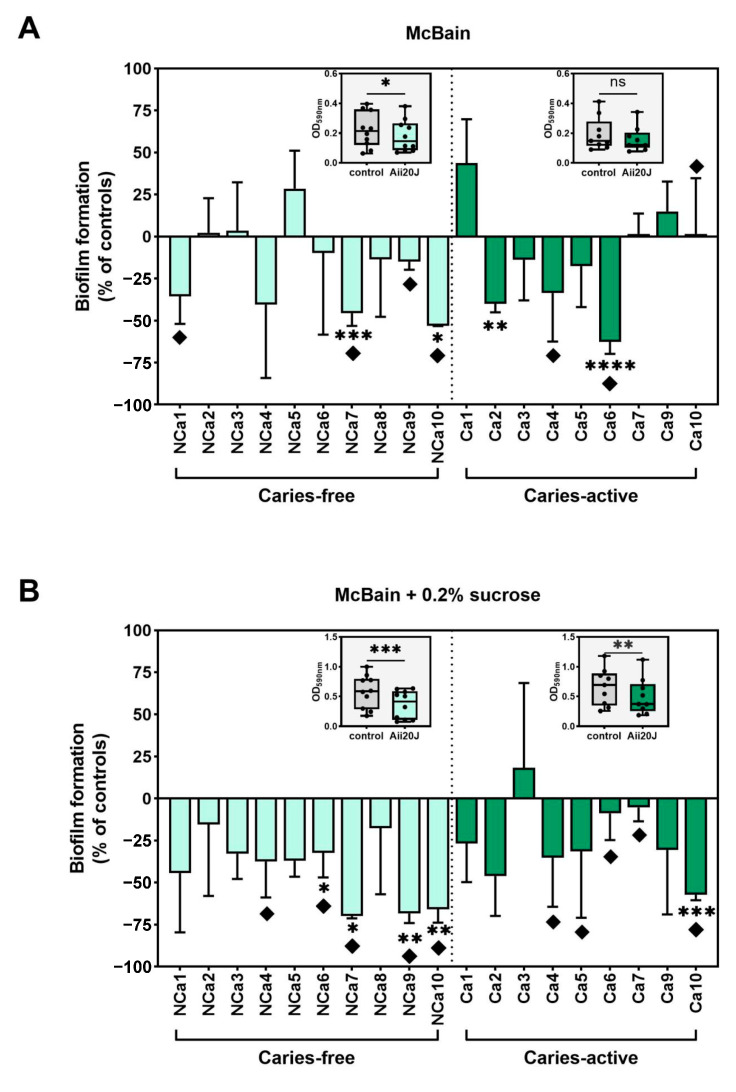
In vitro biofilm formation of supragingival-derived biofilm samples in the presence of Aii20J. Histograms represent the biofilm mass achieved in Aii20J-treated samples as a percentage of the biofilm formed in untreated controls, assessed by CV assay (y-axis). Results are expressed as means, with error bars for standard deviation (*n* = 3). Supragingival biofilm samples used to inoculate the in vitro biofilms were obtained from caries-free (light green bars) and caries-active children (dark green bars). Biofilms were generated in McBain medium (**A**), and McBain supplemented with 0.2% sucrose (**B**). Diamonds (♦) mark macroscopical differences between Aii20J-treated and untreated biofilms. Statistical significance of pairwise comparisons between each Ai20J-treated biofilm and its untreated control is displayed as * *p* ≤ 0.05, ** *p* ≤ 0.01, *** *p* ≤ 0.001, **** *p* ≤ 0.0001 (*t*-tests, α = 0.05). Boxplots are graphed with the median and interquartile range, with whiskers ranging from min. to max. of absolute values of biomass (OD_590nm_) in untreated and Aii20J-treated samples from caries-free (*n* = 10) and caries-active subjects (*n* = 9). Individual values are represented as black dots. Statistical significance of differences in biomass upon Aii20J treatment are marked with asterisks (*) (Wilcoxon tests, ns = non-significant, * *p* ≤ 0.05, ** *p* ≤ 0.01, *** *p* ≤ 0.001).

To evaluate the antibiofilm potential of Aii20J, biofilm mass values obtained in the presence of the enzyme were compared to that of untreated controls. Figure 4A,B displays values of biofilm quantification after treatment with Aii20J, represented as the percentage of biofilm mass achieved relative to untreated controls in the two culture conditions studied. Overall, biofilm mass was reduced in the presence of Aii20J. In biofilms grown in McBain, macroscopical differences were observed in 40% of caries-free biofilms and 33% of caries-active biofilms (Figure 4A). Additionally, statistically significant differences were observed for 20% of caries-free and 22% of caries-active biofilms (*t*-test) (Figure 4A). In biofilms grown in the presence of sucrose (Figure 4B), macroscopical differences were observed in 50% of caries-free and 55% of caries-active biofilms, and statistically significant differences were found in 40% of caries-free and 11% of caries-active biofilms (Figure 4B). The effect of Aii20J on the biofilms was further analyzed by grouping the samples according to the oral health status of the subjects. Wilcoxon tests comparing biomass values of untreated biofilms with biomass values after treatment with Aii20J, revealed statistically significant differences upon Aii20J-treatment in the caries-free biofilms grown in McBain (inserts in Figure 4A), and both the caries-free and caries-active biofilms grown in McBain-sucrose (inserts in Figure 4B). Altogether, these results indicate that biofilms grown in the presence of sucrose respond to Aii20J with greater biomass reductions than those grown without sucrose. The selection of saccharolytic bacteria present in the initial supragingival biofilms is expected with the use of a culture medium supplemented with sucrose. Thus, the presence of Aii20J seems to have had a greater effect on these bacteria than on bacteria selected in the medium without sucrose.

To investigate the microbial composition of the biofilms that responded to the Aii20J treatment with the greatest biomass reductions, gDNA from biofilms grown in McBain-sucrose was extracted, and the 16S rRNA gene was sequenced. Figure S2 shows the most relatively abundant genera identified in the biofilms generated in the presence of sucrose, under exposure to Aii20J, and as untreated controls.

Overall, in vitro biofilms displayed a much lower diversity than the initial supragingival samples. The most abundant genus among the in vitro biofilms was *Streptococcus*, with 86.6–99.9% relative abundance, followed by *Granulicatella* and *Neisseria*, with 0–4% and 0–12% relative abundances, respectively (Supplementary Materials Figure S2). The exceptions to this tendency were biofilms Ca9 and Ca10, which comprised 33–58% *Streptococcus* relative abundance. In these samples, *Veillonella* was identified in a 16–56% relative abundance, and *Neisseria* was found in <0.1–21% relative abundance (Supplementary Materials Figure S2). Overall, biofilms from caries-free samples comprised 2–6 identified genera (samples NCa1–Nca3 and NCa7–Nca10), with some samples reaching up to 24 genera (sample NCa5); whereas, in most caries-active samples, 10–16 genera were identified (samples Ca1, Ca3–Ca5, Ca8–Ca10). When comparing the microbiota composition of untreated biofilms and biofilms exposed to Aii20J, no significant differences were found in caries-free or caries-active groups (Supplementary Materials Figure S2).

**Figure 5:**
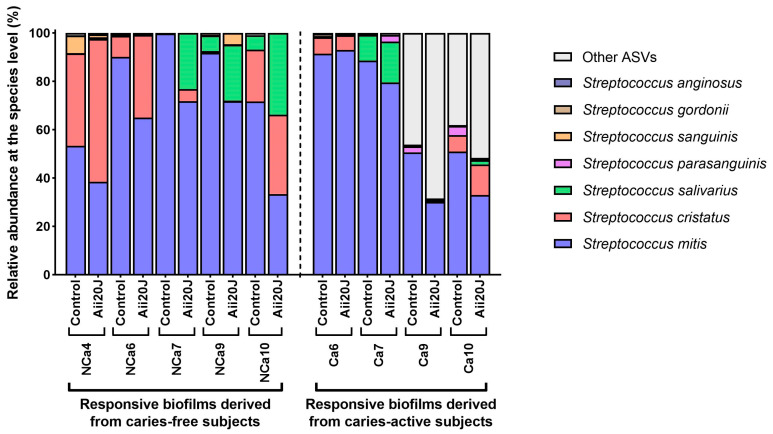
Relative abundance of *Streptococcus* species in in vitro biofilms obtained from supragingival biofilm samples. Full-length 16S rRNA gene sequences were obtained by PacBio sequencing. Histograms show the relative abundance of the species identified in biofilms that were reduced in biomass upon exposure to the enzyme Aii20J.

Further analyses of the microbial composition at the species level were performed on biofilms that responded to the treatment with Aii20J with important reductions in biomass. The 16S rRNA gene of these samples was sequenced by PacBio, and the taxonomical assignment of these full-length sequences was analyzed. Surprisingly, among the amplicon sequence variants (ASVs) belonging to the genus *Streptococcus*, we observed a significant decrease in *Streptococcus mitis* in almost all samples treated with Aii20J (*t*-test, α = 0.05) (Figure 5). In parallel, the relative abundance of *Streptococcus cristatus* and *Streptococcus salivarius* increased in the same samples (Figure 5). Similar results have been reported by Muras et al. [12]; the use of Aii20J on a polymicrobial biofilm dominated by *Streptococcus* spp. resulted in a decrease in the relative abundance of *Streptococcus vestibularis,* while the relative abundance of *Streptococcus oralis* subsp. *dentisani* increased [12]. Notably, the *Streptococcus* species that are increased in biofilms treated with Aii20J are all health-associated, and they have even been proposed as probiotics in the oral field [36,37,38]. In the present study, the probiotic potential of *S. cristatus* and *S. salivarius* derives from the expression of pH-buffering enzymes: arginine deiminase in *S. cristatus* [39] and urease in *S. salivarius* [2]. The production of ammonia that results from the activation of these enzymes can counteract the low pH that characterizes cariogenic environments, pointing towards the biofilm-modulating potential of Aii20J. Interestingly, pure *Streptococcus* cultures are not affected by the QQ enzyme Aii20J [9]. This evidence suggests that *Streptococcus* might interact with intergeneric cues (possibly AHLs or AHL-like molecules) produced by other taxa present in the polymicrobial biofilms and that the function of these intergeneric cues changes upon Aii20J exposure, as suggested in a previous study performed in our laboratory [27]. Additionally, several studies have already pointed out a role of AHLs on gram-positive taxa [40,41].

The in vitro nature of this work allows us to study complex polymicrobial biofilms in a controlled setting. Nevertheless, such an approach inevitably limits the conclusions obtained. Although we describe changes in biofilm formation abilities via biomass quantification methods, our results do not offer insights into the activation of cariogenic metabolic factors within biofilms as studying the differential expression of virulence-related genes could have. Additionally, the translation of these investigations to an in vivo setting would be desirable, as QQ strategies display increasing evidence of their potential for preventing and treating biofilm-related infections. Such in vivo studies are necessary to support the proposal of using QQ in clinical settings, probably as adjuvants to antibiotic treatments to enhance their bactericidal action [42]. Last, studying the potential interactions between AHLs or AHL-like molecules and *S. salivarius* and *S. cristatus* would be interesting to further investigate the mechanisms underlying the changes in their relative abundances.

Altogether, our results show that Aii20J significantly reduces biofilms that are vastly dominated by gram-positive taxa, specifically *Streptococcus*. The observed changes in biofilm behavior are probably due to the surrounding environment of the polymicrobial biofilms generated. These results further support the use of Aii20J as an antibiofilm strategy for the prevention and treatment of oral diseases and are a potential alternative or adjuvant to antimicrobial therapy. Furthermore, our results point towards the potential interactions of gram-positive taxa and intergeneric cues produced in polymicrobial environments.

## 3. Materials and Methods

### 3.1. Subject Recruitment and Ethics Statements

A cohort of ten caries-free children (female/male (F:M): 4:6, mean age: 8.7 ± 3.2 years), and ten caries-active children (F:M: 5:5, mean age: 7.8 ± 1.5 years) were recruited from a dental practice in Santiago de Compostela (ES) between December 2021 and July 2022 (Table 1). The inclusion criterium was the age of the subjects, with eligible children between 5 and 12 years old from those attending the dental practice. Orthodontic appliances and the use of antibiotics up to one month prior to sampling were the exclusion criteria set for this study.

The investigation protocol 2021/495 for patient recruitment and sample handling, modified in November 2021, was approved by the Ethical Committee of Clinical Investigations of Galicia (Xunta de Galicia, ES). Written informed consent was obtained from the legal guardians of all participants.

### 3.2. Sample Collection and Growth Conditions

Supragingival plaque samples were taken from ten caries-free children (codes NCa1–NCa10) and ten caries-active children (codes Ca1–Ca10) (Table 1) by scraping the vestibular and lingual surfaces of four dental pieces with a curette, one in each quadrant of the mouth. The same number deciduous and permanent pieces were sampled when possible. Supragingival plaque was stored in 1.5 mL of thioglycolate medium (Merck Millipore, Burlington, MA, USA) at 4 °C and processed promptly. Samples (*n* = 20) were vortexed for 30 s, and 100 μL were set aside and frozen at −80 °C for gDNA extraction. The remaining volume was diluted 1:53 [43] in either McBain or McBain supplemented with 0.2% sucrose (McBain-sucrose). Samples were grown in the AAA model, and, when necessary, Aii20J was added at 20 µg/mL. Biofilms were grown in aerobiosis at 37 °C for 24 h, refreshing culture media and treatments at 12 h.

### 3.3. Strains and Culture Conditions

*Chromobacterium subtsugae* CV026, formerly *Chromobacterium violaceum* CV026, was used in this study. *C. subtsugae* CV026 is able to respond to AHLs with acyl chains between 4 and 8 carbon atoms in length by producing a violet pigment but does not produce endogenous AHLs [44]. *C. subtsugae* CV026 was routinely plated in Luria Bertani (LB) with 25 μg/mL of kanamycin and grown at 30 °C.

### 3.4. Production and Purification of the QQ Enzyme Aii20J

The QQ enzyme Aii20J, an AHL-lactonase enzyme obtained from the bacterium *Tenacibaculum* sp. strain 20J was obtained as previously described [45]. Briefly, protein expression of the recombinant *E. coli* BL21(DE3)pLysS containing Aii20J was induced when suspensions reached 0.6 OD_600nm_ by the addition of 1 mM Isopropyl-D-thiogalatopyranoside (IPTG) followed by further overnight incubation at 22 °C with gentle orbital shaking. The induced cells were pelleted, resuspended in phosphate buffered saline (PBS), and then lysed by sonication. Imidazole was added at a final concentration of 20 mM to prevent nonspecific binding during later purification steps. Purification of Aii20J was achieved using the His GraviTrap affinity column protein purification kit (GE Healthcare) following the manufacturer’s instructions. Briefly, the columns have a nickel-based medium for the purification of histidine-tagged proteins by immobilized metal affinity chromatography. The first step was the equilibration of the column with a phosphate buffer (10 mM Na_2_HPO_4_·2 H_2_O, 10 mM Na_2_H_2_PO_4_·H_2_O, 500 mM NaCl) containing imidazole in the same concentration as in the sample (20 mM) to prevent binding of host cell proteins with exposed histidines to the nickel-based medium. After sample flow-through, a washing step was performed with the 20 mM-imidazole buffer. Last, elution of the affinity-bound protein was achieved with a 500 mM-imidazole buffer. The remaining imidazole was then removed from the eluted sample by dialysis with D-tubes (MWCO 10 kDa) (Merck Millipore, Burlington, MA, USA) in sterile Milli-Q water.

The QQ enzyme Aii20J was added to the assays to a standardized working titer of 20 µg/mL. The AHL-degradation capacity of the enzyme was routinely verified using chromogenic assays (see Section 3.5).

### 3.5. Quorum Quenching Activity Solid Plate Assay

Solid plate bioassays were used to assess the AHL-degradation activity of the QQ enzyme Ai20J, purified and in biofilm supernatants after incubation. Ten-fold serial dilutions of the purified enzyme were prepared until reaching a titer of 0.02 μg/mL of Aii20J. The established minimum active concentration (MAC) of Aii20J is 2 μg/mL [46]. For biofilm supernatants treated with Aii20J at a titer of 20 μg/mL, 100 μL was transferred to a fresh microtube at the end of the incubation time. Both the purified Aii20J and biofilm supernatants containing Aii20J were incubated with 10 μM of C_6_-HSL for 3 h at 22 °C. PBS (pH 6.5) with 10 μM of C_6_-HSL was used as a negative control [47]. The bioassays were carried out as described elsewhere [27].

### 3.6. Biofilm Generation and Quantification

In vitro biofilms derived from supragingival samples were generated using a modification of the AAA model [26,33], as previously described [27]. Briefly, custom stainless-steel lids for 12-well culture plates (VWR) were used to allow the insertion of glass coverslips (18 × 18 mm) (Menzel Gläser, Braunschweig, Germany) [26,27]. The wells were filled with 3 mL of inocula and, when needed, Aii20J was added to the standardized titer of 20 µg/mL. Sterile Milli-Q water was added to negative control biofilms. After the incubation time, biofilms quantification was performed by CV assay [27]. After CV staining, biofilms were visually examined [27]. Absorbance of CV-stained biofilms was measured at 590 nm in a Multiskan SkyHigh (Thermo Scientific, Waltham, MA, USA). Experiments were performed in triplicate.

### 3.7. Investigation of the Microbial Composition of Biofilms

#### 3.7.1. Genomic DNA Extraction

Genomic DNA for sequencing analysis was obtained from initial supragingival biofilm samples and biofilms grown in the AAA model. For the latter, biofilms were harvested by 15 min-sonication of the glass coverslips in 5 mL of sterile PBS. Genomic DNA extraction was performed using the “DNeasy PowerBiofilm Kit” (Qiagen, Hilden, Germany), following the manufacturer’s instructions [27], and DNA concentration was measured using a Qubit 4 Fluorometer (Thermo Scientific, Waltham, MA, USA).

#### 3.7.2. Library Preparation and Microbiome Analysis

The library preparation and sequencing were performed in the Foundation for the Promotion of Health and Biomedical Research of Valencia Region (FISABIO) (ES). Genomic DNA (5 ng/µL in 10 mM pH 8.5) was used to amplify the V3-V4 hypervariable regions of the 16S rRNA gene. The libraries were prepared according to Illumina’s protocol and sequenced using a 2 × 300 base pair paired-end run on a MiSeq Sequencer. Quality assessment was performed using the prinseq-lite [48]. The analysis and clustering of the sequences into amplicon sequence variants (ASVs) was conducted using the pipeline DADA2 [49]. Classification of the ASVs to the genus level was conducted using the SILVA database [50]. Computations and statistics were performed in R [51] using knitr, knitcitations, markdown, biostrings, and vegan [52,53,54,55,56].

A selection of biofilms that responded to the Aii20J treatment with reductions in their biomass was additionally used to sequence the full 16S rRNA gene in a PacBio Sequel II sequencer. Data derived from the sequencing were obtained using an ad hoc pipeline written in RStatistics environment [51]. Sequence data were analyzed using the qiime2 pipeline [57].

A LDA of the identified taxa was performed with the packages Phyloseq and DESeq2 [58]. Only differences with a log fold-change, either below 2 or above 2, were considered relevant.

### 3.8. Statistical Analyses

Statistical analyses were performed using GraphPad Prism 9.5.1 (GraphPad, San Diego, CA, USA,). The normality of the datasets’ distribution was tested using Shapiro–Wilk tests with an α = 0.05. Two-tailed Student’s *t*-tests (referred to in the text as *t*-tests) for normally distributed samples and Mann–Whitney tests for non-normally distributed samples were performed to determine the statistical significance of the differences between the control and Aii20J-treated biofilms within the same subject. In addition, Wilcoxon tests were performed to assess the significance of the reductions in biofilm formation in the presence of Aii20J throughout samples originated from different subjects and grown in the same conditions. Significant differences were determined with an α = 0.05 for all the statistical analyses.

## Figures and Tables

**Table 1:** Classification of the oral health status of the subjects (*n* = 20). F: female. M: male. N/A: not available.

Code	Age	Sex	Classification
NCa1	3	M	Caries-free
NCa2	11	F	Caries-free
NCa3	13	M	Caries-free
NCa4	11	M	Caries-free
NCa5	N/A	M	Caries-free
NCa6	9	F	Caries-free
NCa7	8	F	Caries-free
NCa8	7	M	Caries-free
NCa9	5	F	Caries-free
NCa10	11	M	Caries-free
Ca1	6	M	Caries-active
Ca2	9	M	Caries-active
Ca3	7	F	Caries-active
Ca4	6	M	Caries-active
Ca5	7	M	Caries-active
Ca6	9	M	Caries-active
Ca7	6	F	Caries-active
Ca8	10	F	Caries-active
Ca9	9	F	Caries-active
Ca10	9	F	Caries-active

## Data Availability

The data presented in this study are available on request from the corresponding author.

## Supplementary Materials

The following supporting information can be downloaded at: https://www.mdpi.com/article/10.3390/antibiotics12081263/s1, Figure S1: Principal components analysis (PCA) plots of the microbial composition at the species level of supragingival biofilms. Figure S2: Relative abundance at the genus level of in vitro biofilms obtained from supragingival biofilm samples.
