# Supplementary material for: New Preventive Strategy against Oral Biofilm Formation in Caries-Active Children: An In Vitro Study
Source: Antibiotics (Basel). 2023 Jul 31;12(8):1263. doi: 10.3390/antibiotics12081263 (PMC10451667; doi:10.3390/antibiotics12081263)
Supplement: Supplementary file 1 [file antibiotics-12-01263-s001.zip › antibiotics-2500603-supplementary.pdf]

Supplementary Materials

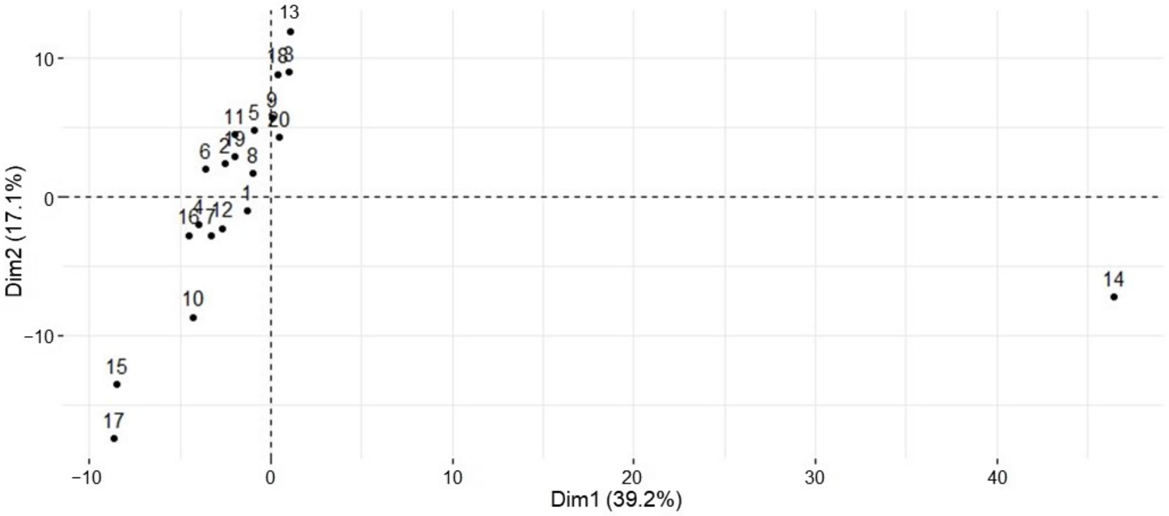

Figure S1. Principal Components Analysis (PCA) plots of the microbial composition at the species level of supragingival biofilms. Supragingival biofilms were collected with curette from caries-free and caries-active children (n = 20).

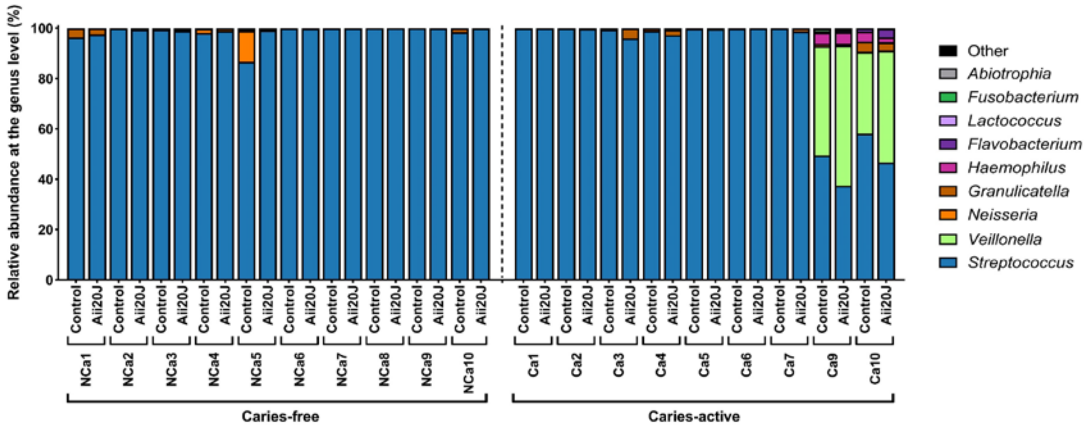

Figure S2. Relative abundance at the genus level of *in vitro* biofilms obtained from supragingival biofilm samples. Supragingival samples were collected with curette from caries-free (NCa) and caries-active children (Ca). Biofilms were generated in the AAA model and exposed to Aii20J or left as untreated controls. Relative abundance represented as the percentage of the most abundant genera identified in each sample (y-axis). Genera detected in lower relative abundance were grouped in the category "Other".
